# The relationship between patellofemoral arthritis and fat tissue volume, body mass index and popliteal artery intima-media thickness through 3T knee MRI

**DOI:** 10.3906/sag-1811-166

**Published:** 2019-06-18

**Authors:** Mehmet KURT, Ali Yusuf ÖNER, Murat UÇAR, Seda ALADAĞ KURT

**Affiliations:** 1 Department of Radiology, Van Lokman Hekim Private Hospital, Van Turkey; 2 Department of Radiology, Faculty of Medicine, Gazi University, Ankara Turkey; 3 Department of Radiology, Van Training and Research Hospital, Van Turkey

**Keywords:** Chondromalacia patella, infrapatellar fatpadvolume, body mass index, patellar cartilage volume, 3T MRI

## Abstract

**Background/aim:**

Evaluating the relationship of patellar chondromalacia with obesity, infrapatellar fat pad (IFP) volume and popliteal artery intima-media thickness (IMT).

**Materials and methods:**

A total of 203 patients with different degree of patellar chondromalacia (103 male, 100 female) and 52 control subjects (19 male, 33 female) were included and grouped according to sex, age, body surface area (BSA), body mass index (BMI) and patellar chondromalacia classification. All measurements were completed with 3T magnetic resonance imaging (MRI). Articular cartilage and IFP volume were measured in saggital plane using double echo steady state (DESS) and DIXON sequences, respectively. Patellar cartilage damage was graded using modified outerbridge classification, and the relations among cartilage volume and BMI, BSA, IFP, IMT were statistically assessed.

**Results:**

Popliteal artery IMT showed an independent association with the prevalence of cartilage defects and IFP volumes (P ˂ 0.001). There was an association between BMI and IFP volumes (P ˂ 0.001). However, no differences were observed between IFP volume and different chondromalacia groups. When IFP measurements were corrected using individual BMI and BSA values, a positive correlation was found between control and advanced chondromalacia groups (P ˂ 0.001).

**Conclusion:**

This study demonstrates the relationship among obesity, IMT and chondromalacia and highlights this potential circle to develop effective treatments and inhibit the progression of chondromalacia.

## 1. Introduction

Degenerative joint disorders like patellofemoral arthritis are leading causes of morbidity and economic loss, and have a significant impact on community health in the adult population. In addition to biomechanical causes, a multifactorial interaction based on specific roles of some inflammatory and vascular causes has also been suggested in the etiology, with particular focus in recent years being on infrapatellar fat tissue and vascular wall changes [1,2].

There have been a limited number of studies to date, reporting increased infrapatellar fat tissue volume, and a correlation between body mass index (BMI) and infrapatellar fat tissue volume in patients with osteoarthritis (OA) [3]. There are studies investigating vascular wall characteristics, and reporting a positive correlation between popliteal artery wall thickness and OA [4].

Also, it has been reported that there is a significant relationship between visceral and subcutaneous fat tissue and atherosclerosis and that these fat tissues are metabolic risk factors [5,6]. Recent studies into the physiology of fat tissue have suggested that destruction of the joint cartilage seen in overweight people may also be associated with some systemic factors, aside from mechanical load. Studies have indicated that the infrapatellar fat pad (IFP) releases inflammatory mediators, and plays a significant role in the onset and progression of OA [3,7].

In the present study, we evaluated the relationship among obesity, IFP volume, patellar cartilage volume (PCV), and popliteal artery intima-media thickness (IMT) through the use of 3T magnetic resonance imaging (MRI) in adult patients with patellofemoral arthritis.

## 2. Materials and methods

This study included a total of 255 subjects (mean age: 44 years, range: 20–65 years) who presented with various complaints of the knee joint and who were referred for an MRI with different preliminary diagnoses. Subjects with bilateral knee MRIs, a history of surgery, and movement artifacts were excluded from the study. After the Institutional Review Board approved, informed consent was obtained from all patients. The study was approved by the Ethics Committee of Gazi University Faculty of Medicine (Date: 16/11/2011, decision number: 338).

The subjects were classified into two groups, being those with preliminary diagnosis of arthritis as the ‘patient’ group, who had clinical symptoms for arthritis, and those with meniscus and/or ligament pathologies who were assigned as ‘control’ group. The control group was defined as subjects with normal body mass index and no cartilage pathology. Although the subjects were reported as grade 0 chondromalacia after imaging, they were considered as ‘patient’, because of having arthritis findings. Table 1 presents the data on patient and control groups, related to BMI according to subgroups as ‘normal’ for group A, ‘overweight’ for group B and ‘obese’ for group C. And Table 2 presents the age distribution according to the stage of chondromalacia.

**Table 1:** Age distributions of subgroups according to BMI.

	Patient Group			Control Group
	Group A (Normal)	Group B (Overweight)	Group C (Obese)	
Women	51.33 ± 10.95 (n = 16)	47.47 ± 10.20 (n = 52)	50.32 ± 8.19 (n = 32)	32.47 ± 11.94 (n = 33)
Men	51.57 ± 17.99 (n = 16)	43.78 ± 12.51 (n = 59)	41.85 ± 10.29 (n = 28)	35.05 ± 13.89 (n = 19)

n: The number of patients.

**Table 2:** Age distributions of cases according to outerbridge classification.

	Grade 0	Grade 1	Grade 2	Grade 3	Grade 4
Women	39.81 ± 13.27(n = 67)	44.60 ± 9.09 (n = 10)	44.82 ± 8.08(n = 17)	54.07 ± 9.32 (n = 14)	53.72 ± 7.08(n = 29)
Men	39.03 ± 13.87(n = 64)	38.96 ± 10.03 (n = 25)	50.11 ± 8.31 (n = 9)	61 ± 10.52 (n = 4 )	55.19 ± 7.42(n = 16)

n: The number of patients.

Knee MRI images were obtained using 3T superconducting magnets (Siemens Magnetom Verio, Erlangen, Germany) and standard 8-chanelled knee bandages, while the patients were in the supine position. Table 3 lists the sequences and parameters used during the imaging process.

**Table 3:** Acquisition parameters used in the knee MRI.

Parameters	PD FAT SAT	T2 FAT SAT	PD FAT SAT	T2	T1	DIXON	DESS
	Axial	Coronal	Sagittal	Sagittal	Coronal	Sagittal	Axial
Section Thickness (mm)	4	4	4	4	4	4	0.80
Section number	25	25	25	25	25	96	112
FOV(AP)	160	160	160	160	160	254	192
Acquisition matrix	384 x 326	448 x 358	384 x 288	448 x 336	384 x 288	288 x 209	320 x 320
TR (ms)	2800	4230	3800	4000	550	7,46	14.52
TE (ms)	34	80	34	90	17	2.45/3.67	5.04
Average (NEX)	2	2	2	1	1	2	1

After the digital images were transferred from the archive to the work station, the images were evaluated in a single session by two radiologists, who recorded such data as age, sex, BMI, and body surface area (BSA) for each subject, after which, measurements of infrapatellar fat tissue volume, PCV and popliteal artery wall thickness were obtained.

**Figure 1:**
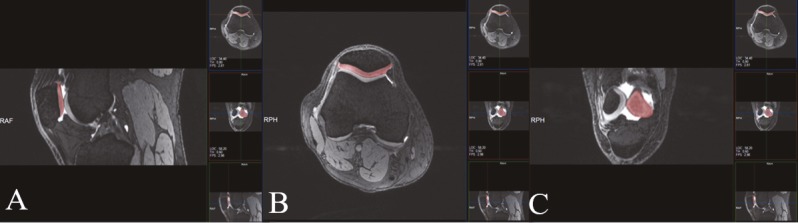
Drawing of the borders of cartilage in the sagittal (A), axial (B), and coronal (C) planes while calculating volume in a sample of control group.

Based on the BMI, the cases were classified as normal weight (20.0–24.99 kg/m²), overweight (25.0–29.99 kg/m²), and obese (30.0–39.99 kg/m²). The level of cartilage destruction in the cases was evaluated using a 3D DESS sequence and classified based on the modified outerbridge cartilage destruction grading system [8,9] (Table 4). Cartilage borders over a same sequence were marked interactively and at all three dimensions using an interactive manual software program on the ‘Extreme PACS’ work station, and the knee joint cartilage volume for each case was calculated (Figure 1). Cartilage borders were marked based on the criteria defined by Baysal et al. [10], and were measured at all three planes, with the sagittal plane being more pronounced until bone-cartilage separation, and separately including both the patellar and femoral cartilage borders. The total cartilage volume was calculated by the software by multiplying the total measured area with the slice thickness.

**Table 4:** Modified outerbridge classification.

Grade	Macroscopy	MRI
Grade 0	Normal cartilage	Normal cartilage
Grade 1	Rough surface; chondral softening, focal thickening	Inhomogenous, high signal; surface intact, cartilage swelling
Grade 2	Irregular surface defects; <50% of cartilage thickness	Superficial ulceration, fissuring, fibrillation; <50% of cartilage thickness
Grade 3	Loss of >50% of cartilage thickness	Ulceration, fissuring, fibrillation; >50% of the depth of cartilage
Grade 4	Cartilage loss	Full thickness chondral wear with exposure of subchondral bone

**Figure 2:**
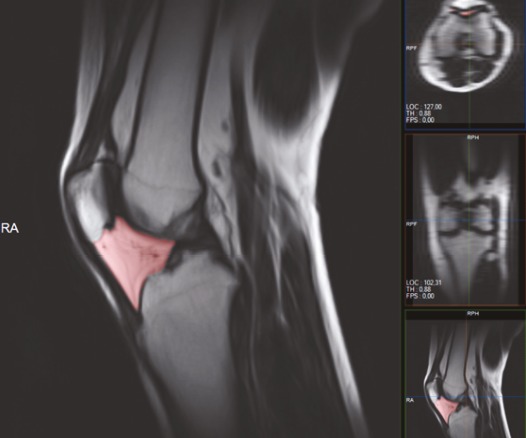
Drawing the borders of IFP in the sagittal plane while calculating the volume in a sample of control group.

Infrapatellar fat pad measurements were obtained using the 3-point Dixon sequence in the Extreme PACS system using software that allowed interactive manual drawings (Figure 2). The advantages of the Dixon sequence over other methods include stronger fat suppression, the provision of fat/water imaging, its utilization in quantitative applications, and its high signal-to-noise ratio (SNR) rate [11–15]. The borders of the IFP were marked using the criteria defined by Chuckpaiwong et al. [7].

**Figure 3:**
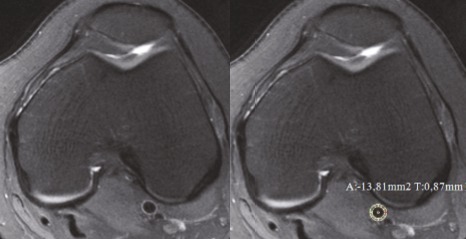
Popliteal artery IMT measurement in the axial plane in a sample of control group.

Popliteal artery IMT measurements were obtained from fat-suppressed axial proton density images. In order to increase wall clarity and measurement sensitivity in this sequence, cardiac synchronization was carried out using a peripheral trigger. IMT measurements were calculated using the Extreme PACS system, with the help of ‘regions of interest’ (ROIs) located on the outer border of the vascular wall. Semi-automated software was used to subtract the lumen volume from the total vascular volume, and the mean wall thickness was obtained taking into account the minimum and maximum values recorded from different sections of the vascular wall (Figure 3). 

**Figure 4:**
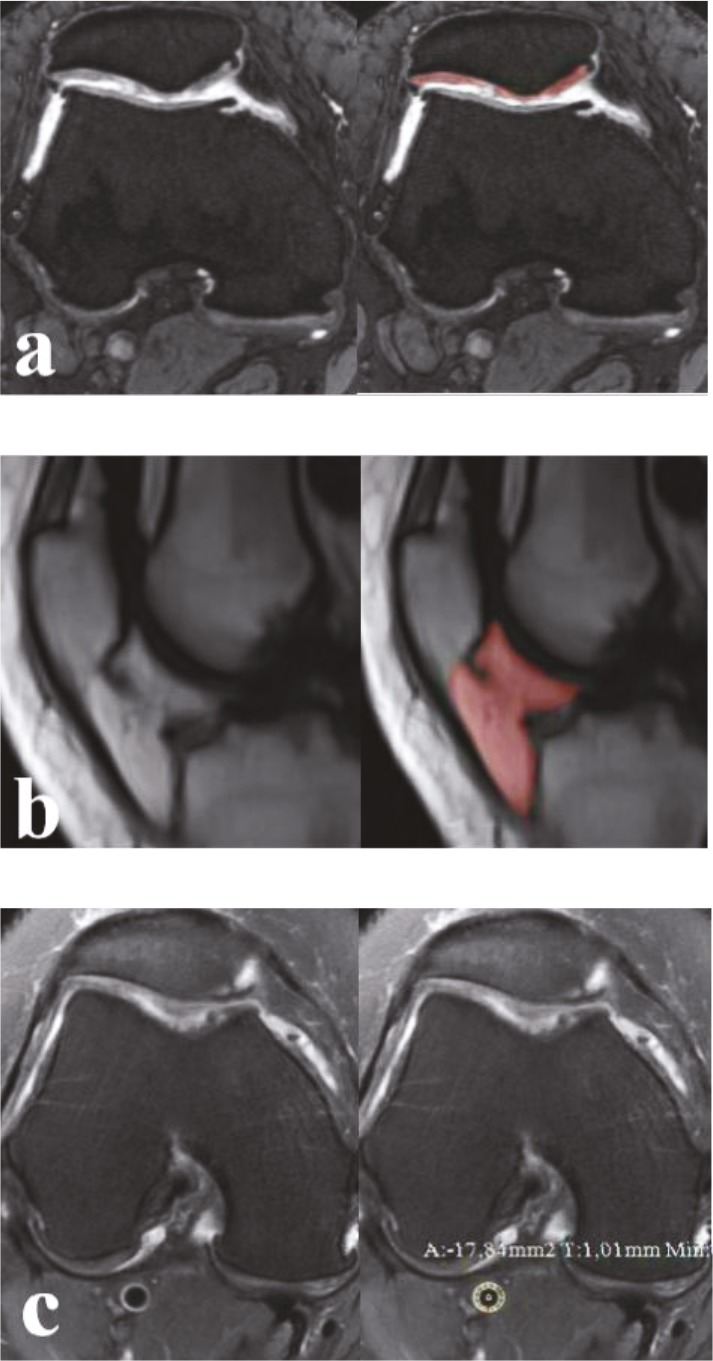
A 48 year-old woman with grade 3 chondromalacia. a. Patellar cartilage volume measurement in DESS sequence in the axial plane. b. IFP measurement in DIXON sequence in the sagittal plane. c. Popliteal artery IMT measurement in PD FAT-SAT sequence in the axial plane.

These measurements were shown on a patient with grade 3 chondromalacia, in Figure 4.

The statistical analysis was carried out using SPSS version 15 (IBM Corp, Armonk, NY, USA) software. The one-way ANOVA test was used to compare the relationships between two groups based on BMI and the modified outerbridge classification, and the Levene’s test was used to evaluate the homogeneity of variances. A P value of <0.05 was considered to be statistically significant. In the event of a significant difference being noted between the groups, binary posthoc comparisons were made using the Tukey’s test. Correlation coefficients were calculated and the statistical significance of any correlations between the groups was investigated with the Pearson’s test.

## 3. Results

The study was carried out on 255 cases, including 122 men (48%) with a mean age of 42.8 ± 13.65 years, and 133 women (52%) with a mean age of 45.18 ± 12.46 years.

**Figure 5:**
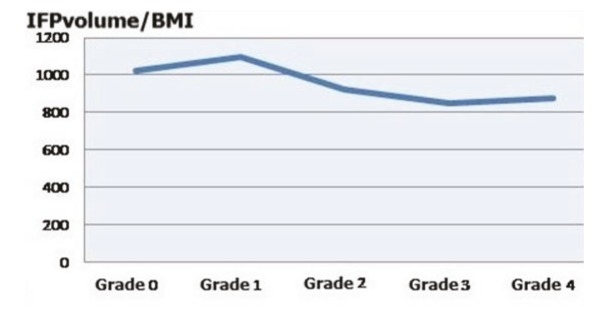
IFP volume/BMI ratios in different chondromalacia grades.

The mean IFP volume in the control and patient groups was 25,130 cm³ and 26,860 cm³, respectively, and the difference between two groups was significant (P ˂ 0.001). When the patients included in the study were classified based on BMI, A as normal, B as overweight and C as obese, the IFP volume in the A, B, and C groups are found to be 24,069 cm³, 26,065 cm³ and 29,737 cm³, respectively. A significant difference was noted between the control group and the C group (P ˂ 0.001). Based on the grade of chondromalacia, the IFP volume in grade 1 cases was significantly higher than in the grade 0 and grade 2 cases (P ˂ 0.001), and there was no significant difference between the other groups. When the relationship between IFP volume and BMI was analyzed, no significant difference was noted between the grade 0 and grade 1 or 2 cases, while the differences between the other groups were significant (P ˂ 0.001) (Figure 5).

**Figure 6:**
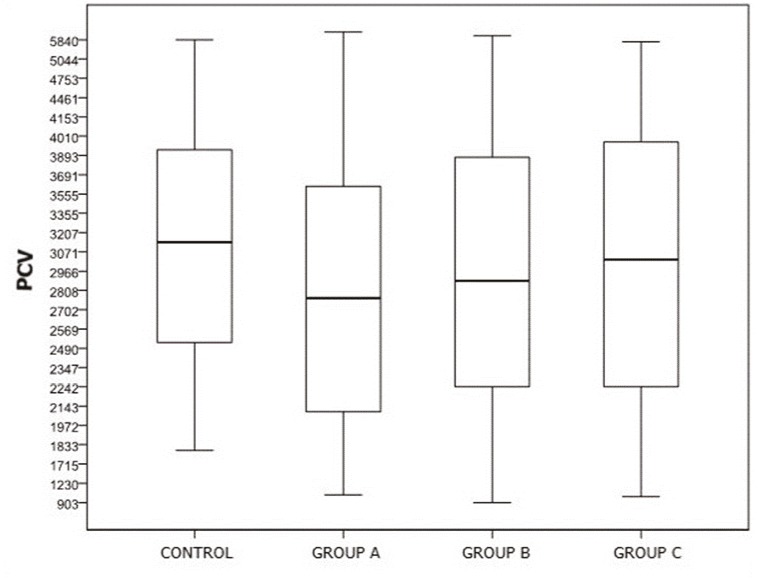
Comparison of PCV averages of patient and control groups.

**Figure 7:**
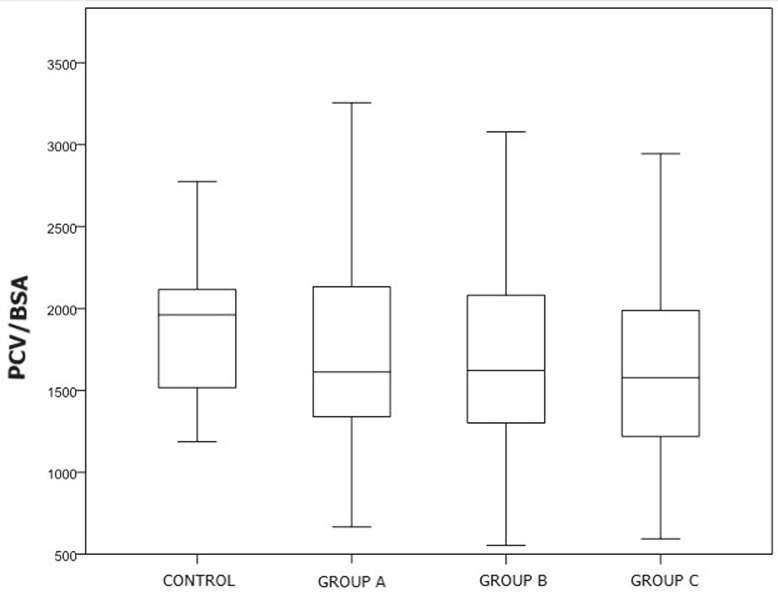
Comparison of PCV/BSA ratios of patient and control groups.

**Figure 8:**
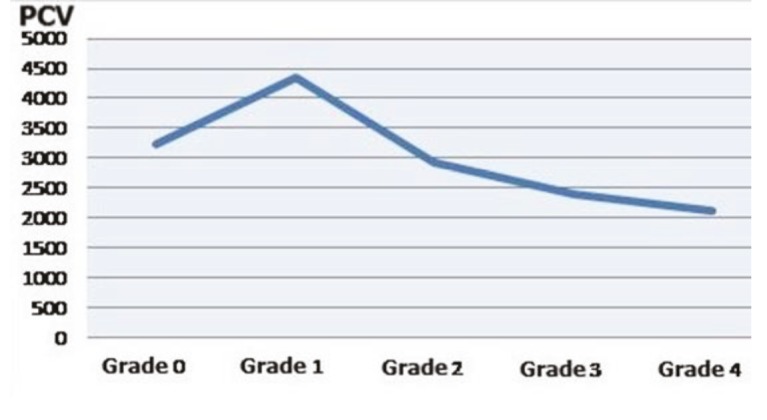
Distribution of PCV in different chondromalacia grades.

A comparison of PCVs uncovered no significant difference between the control group and overall patient population or the subgroups (P ˃ 0.05) (Figure 6). On the other hand, when PCV was evaluated based on BSA, the differences between the groups were not found to be significant, aside from between the control group and group A (P ˃ 0.05) (Figure 7). According to the chondromalacia stages, the average cartilage volume values were 3237 cm³, 4356 cm³, 2925 cm³, 2400 cm³, and 2109 cm³, respectively (Figure 8). The mean PCV decreased as the grade of chondromalacia increased after grade 1. The volume elevation observed in grade 1 was associated with edema observed in cartilage at this stage.

**Figure 9:**
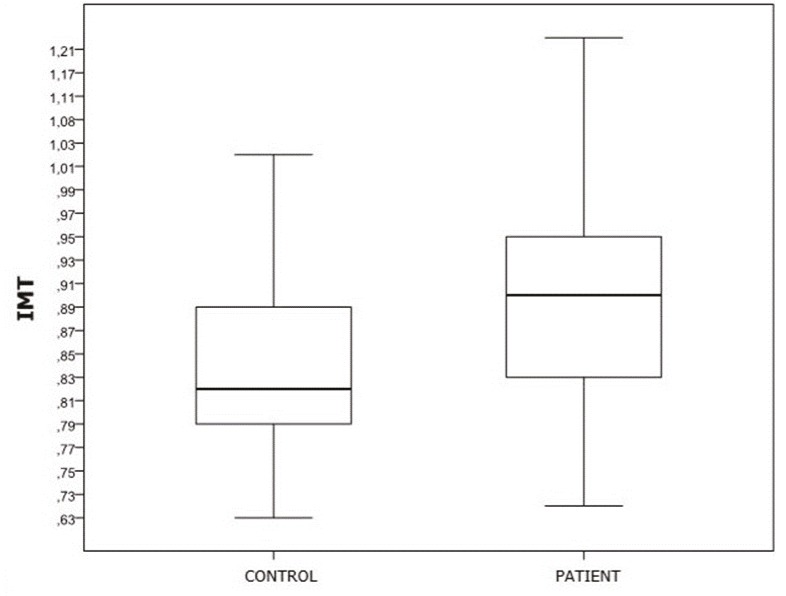
Comparison of popliteal artery IMT values of patient and control groups.

When the mean popliteal artery IMT measurements were compared, statistically significant differences were noted between the control group and all of the patient groups (P ˂ 0.001) (Figure 9). When classified based on cartilage destruction, the mean popliteal artery IMT in the grade 3 and 4 cases were significantly different to those of the other groups (P ˂ 0.001). Furthermore, significant negative correlations were noted when the mean popliteal artery IMT of all groups were compared with the PCV and PCV/BSA ratio (P ˂ 0.001).

## 4. Discussion

Patellofemoral arthritis is the most common form of joint disorder, and is a significant cause of socio-economic loss, and so it would appear to be crucial to clarify the etiopathogenesis of patellofemoral arthritis and to define appropriate treatment approaches [16]. Currently, OA is considered to be a dynamic disease process that develops as a result of an imbalance between the destruction and repair of joint cartilage and the subchondral bone, and this process is possibly influenced by certain metabolic and systemic factors. The treatment goals include the elimination of synovial inflammation and the prevention of cartilage destruction, along with subchondral bone remodeling and gene therapy [1,17].

It has been known for years that obesity represents a risk factor for OA. The Framingham trial has demonstrated that radiography can predict the development of knee OA 36 years before in patients with a BMI of 30 and above [18]. In a study by Lementowski et al. [19], it was found that every 5 kg of weight gain increases the risk of developing OA by 36%, and in a similar study, Coggon et al. [20] reported that the risk of developing OA increased 6.8-fold in individuals with a BMI over 30 when compared to the overall population. While our findings were consistent with literature, the relationship between obesity and chondromalacia in particular was more apparent when cartilage volume was compared based on BSA.

In a study performed by Kok et al. [21], an apparent relationship was reported between chondromalacia patella and subcutaneous fat tissue of the knee in 170 subjects, and women with advanced stages of chondromalacia were found to have thicker subcutaneous fat tissue. In another study, Clockaerts et al. [3] stated that the IFP was a special form of adipose tissue, and played a significant role in the onset of inflammation leading to chondromalacia, being in close proximity to cartilage tissue and synovial surfaces. Some recent studies in the literature support that IFP volume has a protective role for cartilage degeneration. Low IFP values are associated with lower PCV values and increase the risk of cartilage pathologies resulting in osteoarthritis [2,22–24]. In the present study, the grade of chondromalacia had been increasing, as the IFP volume decreased. A significant difference was noted between the patient and control groups when the subjects were classified based on BMI, and the mean IFP volume in obese patients was found to be correlated with an increased BMI. In addition, a significant difference was noted with advancing grades of chondromalacia when the IFP volume/BMI ratios were analyzed based on different grades of chondromalacia. In our study, all of these findings are being based on a large patient population, which contributes significantly to literature.

In a study carried out by Teichtahl et al. [25], obesity was found to increase the risk of cartilage pathologies in both women and men, and PCV in women was found to be associated with increase in the degree of obesity. During the 10-year follow-up in another study by Teichtahl et al. [26], the rate of patellar cartilage defects was found to increase with increasing BMI values. 

In the present study, the effects of increased BMI on PCV were found to be not correlated at the beginning of the study and; therefore, the rating was made based on BSA. This is probably because PCV may vary, depending on BSA in cases with normal BMI. Accordingly, as the weight gain increased, the cartilage volume was decreased.

Several studies have highlighted the negative effects of obesity on vascular structure and function, and there is a multifactorial mechanism that underlies these effects [27]. An increase in body fat ratio alone is a risk factor for atherosclerosis, while an increased intima media thickness is generally considered to be an early sign of atherosclerosis. It is a striking finding that hand OA, seen often in elderly women, is linearly correlated with the degree of atherosclerosis in the individual [28]. This finding points to a relationship between OA and atherosclerosis, irrespective of the factors assumed to be associated with obesity-related over-utilization. In this regard, it is no surprise that factors such as oxidative stress, endothelial dysfunction, and leptin dysregulation can be indicators for OA lesion.

There have been a limited number of studies suggesting that atherosclerosis plays a role in the progression of OA [4]. In a long-term study reported by Hoeven et al. [29] including 2372 men and 3278 women, a relationship was noted between carotid artery IMT and OA of the knee and hand in women. In their study involving 42 patients with OA and 27 controls, Kornaat et al. [4] identified a marked increase in popliteal artery IMT in the group with OA.

In the present study, when the control group was compared with the patient groups that were classified according to BMI, a marked increase was noted in popliteal artery IMT that was found to be correlated with an increase in BMI values, indicating a relationship between obesity and earlyphase atherosclerosis. Popliteal IMT is significantly higher in advanced chondromalacia. Similarly, a negative correlation was noted that as popliteal artery IMT increased, the cartilage volume was decreased. These findings support the potential role of vascular pathology in the onset or progression of OA.

This study has some limitations. The patients analyzed in the study were only symptomatic, and the control group consisted of cases with preliminary diagnoses other than arthritis. As the findings in these patients were not correlated with the findings of any other method, such as arthroscopy or surgery, MRI was considered to be the only appropriate diagnostic tool for the diagnosis of joint cartilage destruction. The control group in this study comprised a relatively small sample of young individuals with a higher mean height than the general population. Accordingly, some ratios were adjusted during the statistical analyses. The findings of the current study indicate a close relationship between obesity and atherosclerosis, although these findings would be further supported by studies involving larger patient populations with a narrower age range, and a simultaneous evaluation of ethnic origin and BMI values, along with an investigation of molecules that are known to be released only from adipose tissue.

**In conclusion, **chondromalacia is a common orthopedic condition in adults, and is the leading cause of patellofemoral pain syndrome. The present study identifies a relationship between obesity, IFP volume, popliteal artery IMT, cartilage volume, and chondromalacia. It is important to further investigate this potential cycle in order to prevent and to come up with an effective treatment for patellar chondromalacia.
